# Gender-Related Differences in Heart Failure Biomarkers

**DOI:** 10.3389/fcvm.2020.617705

**Published:** 2021-01-05

**Authors:** Germán Cediel, Pau Codina, Giosafat Spitaleri, Mar Domingo, Evelyn Santiago-Vacas, Josep Lupón, Antoni Bayes-Genis

**Affiliations:** ^1^Heart Institute, Hospital Universitari Germans Trias i Pujol, Badalona, Spain; ^2^Department of Medicine, CIBERCV, Autonomous University of Barcelona, Barcelona, Spain

**Keywords:** biomarker, heart failure, gender, troponin, natriuretic peptide, ST2, Galectine-3

## Abstract

Important differences in comorbidities and clinical characteristics exist between women and men with heart failure (HF). In particular, differences in the kinetics of biological circulating biomarkers—a critical component of cardiovascular care—are highly relevant. Most circulating HF biomarkers are assessed daily by clinicians without taking sex into account, despite the multiple gender-related differences observed in plasma concentrations. Even in health, compared to men, women tend to exhibit higher levels of natriuretic peptides and galectin-3 and lower levels of cardiac troponins and the cardiac stress marker, soluble ST2. Many biological factors can provide a reliable explanation for these differences, like body composition, fat distribution, or menopausal status. Notwithstanding, these sex-specific differences in biomarker levels do not reflect different pathobiological mechanisms in HF between women and men, and they do not necessarily imply a need to use different diagnostic cut-off levels in clinical practice. To date, the sex-specific prognostic value of HF biomarkers for risk stratification is an unresolved issue that future research must elucidate. This review outlines current evidence regarding gender-related differences in circulating biomarkers widely used in HF, the pathophysiological mechanisms underlying these differences, and their clinical relevance.

## Introduction

Heart failure (HF) is a major health care issue in both sexes; it is associated with significant morbidity, mortality, and health care costs (1). Several differences between women and men have been observed in HF, including the epidemiology, etiology, pathogenesis, risk factors, and prognosis (2). The incidence of HF also differs between men and women, depending on the study population analyzed (3, 4). For example, women had a lower risk of incident HF than men, in middle-aged to older individuals, but women had a higher HF risk than men in the oldest age groups (5). Men tended to be at higher risk of developing HF with reduced ejection fraction (HFrEF), and conversely, women were more likely to develop HF with preserved ejection fraction (HFpEF) (6). This distinction might be attributable to the predisposition of women to develop coronary microvascular dysfunction/endothelial inflammation and the predisposition in men to develop macrovascular coronary artery disease and myocardial infarction (7). These sex-related differences in HF phenotypes and underlying pathophysiology are also reflected in HF biomarker dissimilarities.

In 2007, the National Academy of Clinical Chemistry and the International Federation of Clinical Chemistry recommended the development of sex-specific reference ranges for cardiac biomarkers used routinely in clinical practice (8). Consequently, over the years, sex-driven differences in both reference and cut-off values have been described for several biomarkers in cardiovascular disease (9). However, most of these cardiovascular biomarkers are used day-to-day by clinicians without taking sex into account. It is hypothesized that the lack of sex-specific thresholds for cardiac biomarkers might contribute to under-diagnosing HF in women, which could potentially result in worse outcomes (10).

**Figure 1:**
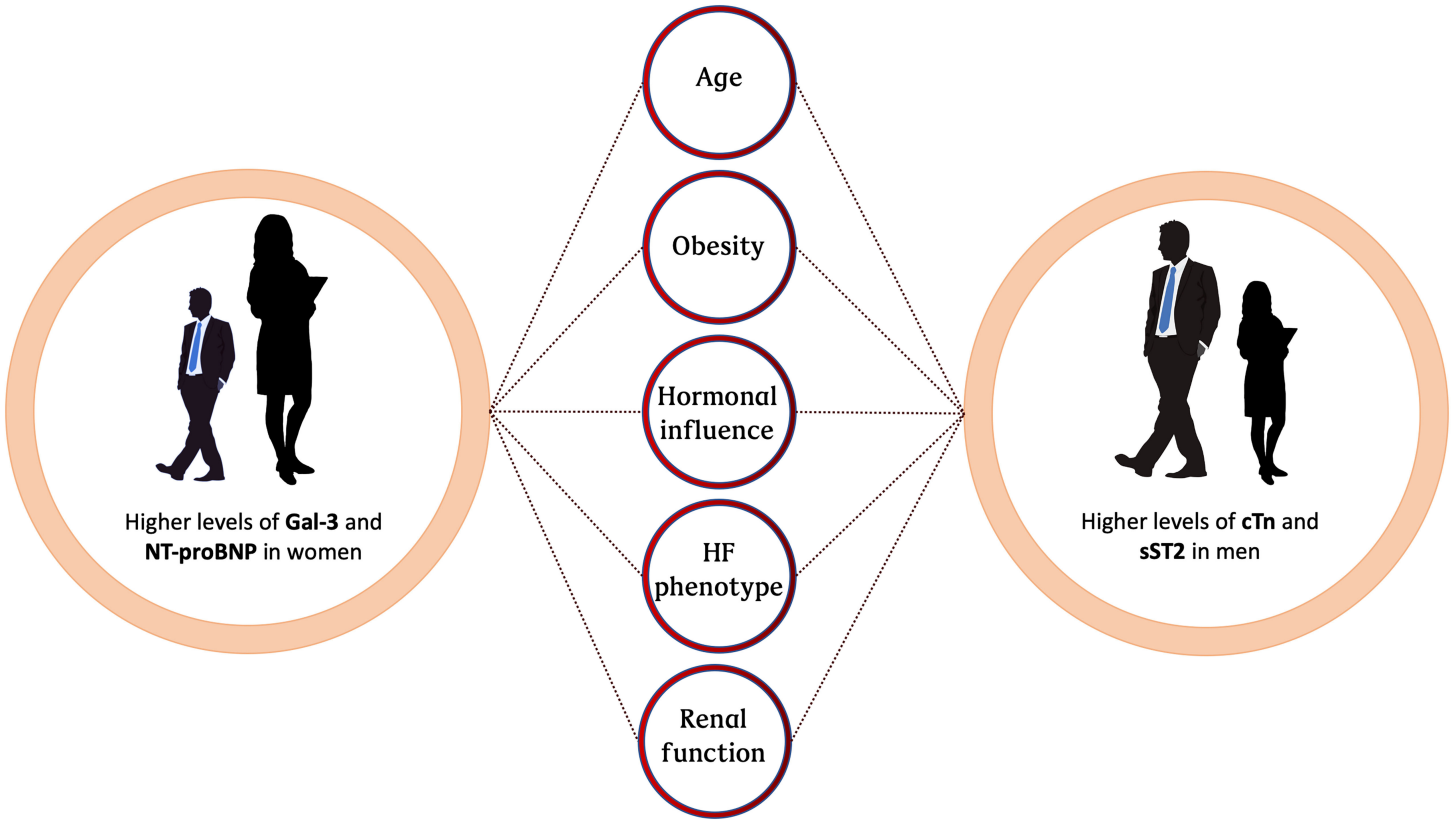
Schematic of factors contributing to sex-related differences in HF biomarkers. HF, heart failure; Gal-3, Galectine 3; NT-proBNP, N-terminal pro-B-type natriuretic peptide; cTn, cardiac troponin; sST2, soluble interleukin-like receptor-like-1.

Improving HF care requires consideration of all gender-related differences. Moreover, improving our understanding of gender-specific differences in HF biomarkers might enrich our understanding of physiological differences between men and women with HF. Taking these points into consideration, this review covers the four most important and frequent HF biomarkers available in daily clinical practice, with a focus on differences between women and men (Figure 1).

## Cardiac Troponin

Currently, assays are available for detecting cardiac troponin (cTn) with high clinical sensitivity and high specificity for myocardial tissue. Moreover, many assays are capable of early cTn detection, when necrosis is minimal or even in the absence of cell necrosis by different mechanisms (increased myocyte turnover or increased cell wall permeability among others). Due to these features, cTn has become the standard biomarker for myocardial damage and the preferred biomarker for diagnosing acute myocardial infarction. In addition, individuals in the HF population frequently have increased concentrations of high-sensitivity cTn (hs-cTn). In up to 93% of patients with acute HF and up to 74% of patients with stable chronic HF, hs-cTn concentrations are above the 99th percentile of the reference value (11). However, several studies and critical reviews have examined sex-related differences in cTn levels that might affect diagnostic and prognostic performance.

### Variations in cTn Concentrations According to Gender

Marked variations in cTn concentrations have been detected between women and men, with higher values commonly found in men (12, 13). This difference has also been evident in patients with HF (14, 15). Consequently, when interpreting cTn results, sex-related peculiarities in the pathobiology of cardiac disease must be considered. Men tend to have a greater cardiac mass and a higher incidence of subclinical coronary artery disease than women (16, 17). Women tend to show less severity in atherosclerosis, left ventricular hypertrophy, and cardiomyocyte apoptosis than men (18, 19). In addition, HFrEF (from ischemic and non-ischemic etiologies) occurs more frequently in men than in women, and HFpEF is more prevalent among women than among men (6, 20). The possibility of an indirect hormonal influence should also be considered, in light of cardioprotective effects of estrogens, which suppress cardiomyocyte apoptosis, and the potentially harmful effects of testosterone, which induces hypertrophy and apoptosis in cardiomyocytes (21–23). Obesity was also independently associated with a positive, linear increase in the likelihood of high hs-cTn levels, as shown in a recent population-based study of subjects without cardiovascular disease at baseline. In that study, individuals with severe obesity and high hs-cTn levels had a >9-fold higher risk of incident HF compared to individuals with normal weight and undetectable hs-cTn levels (24). All these variations could contribute to sex-related differences in serum cTn concentrations and had allowed the thoroughly study of sex-tailored cut-off values of hs-cTn in the setting fundamentally of ACS, where sex-specific cut-off points might improve sensitivity for diagnosis of myocardial infarction in women (25). Diagnostic performance of hs-cTn for HF is however limited. In the general population, the application of dichotomous cut-off values of hs-cTn, lower in women than men: 4.7 vs. 7.0 pg/ml, respectively, for hs-cTnI as studied by Zeller et al., allowed substantial reclassification information for prediction of cardiovascular disease, including HF, being considered an independent predictor of cardiovascular events (26).

### Prognostic Utility of cTn in HF

In the HF spectrum, the diagnostic utility of cTn is limited; however, its prognostic value is highly relevant. Studies by Parikh et al. (27) and by de Boer et al. (28) demonstrated that cTn levels could predict incident HF in different community-based cohorts. Recently, a meta-analysis that pooled data from 16 prospective studies and included nearly 67,000 subjects demonstrated a strong association between cTn and the development of incident HF, and this association was found in both men and women (29). Robust evidence from a meta-analysis based on individual patient data from 10 studies and 11 cohorts (30) also suggested that cTn could become an affordable biomarker for risk stratification in patients with HF, due to the similarity of its prognostic value between men and women. However, data are inconsistent as to whether the prognostic value of cTn differs with sex. Current evidence has indicated that the 99th percentile cutoff values were higher in males than in females (26, 31). However, despite the widespread use of cTn in clinical practice, all available assays lack sex-specific reference values.

## Natriuretic Peptides

Natriuretic peptides are a group of neurohormones that play a central role in the regulation of electrolytes and water balance through their diuretic and natriuretic effects (32). In humans, mainly three forms of natriuretic peptides are found: A-type natriuretic peptide (ANP), B-type type natriuretic peptide (BNP), and C-type natriuretic peptide (CNP). CNP is primarily produced in vascular endothelial cells; ANP and BNP are mostly found in the myocardium. Natriuretic peptides are released by the myocardium in response to stretch and hypoxic stimuli (33). The majority of clinical evidence on natriuretic peptides in the setting of HF is related to BNP and the amino terminal of the proBNP molecule (NT-proBNP). Therefore, this review focuses on NT-proBNP, because it is the best choice for a diagnostic and prognostic biomarker in HF, according to the 2016 European Society of Cardiology HF clinical guidelines (34).

The most extensive evidence on the value of BNP-related *in vitro* diagnostic tests was published in the early 2000s. Comparative studies that measured concentrations of the active BNP hormone vs. NT-proBNP generally demonstrated diagnostic equivalency for differentiating HF from other causes of shortness of breath. The proBNP molecule contains 108 amino acids. The first 76 amino acids are biologically inactive, and amino acids 77–108 constitute the biologically active component of the molecule, BNP.

**Figure 2:**
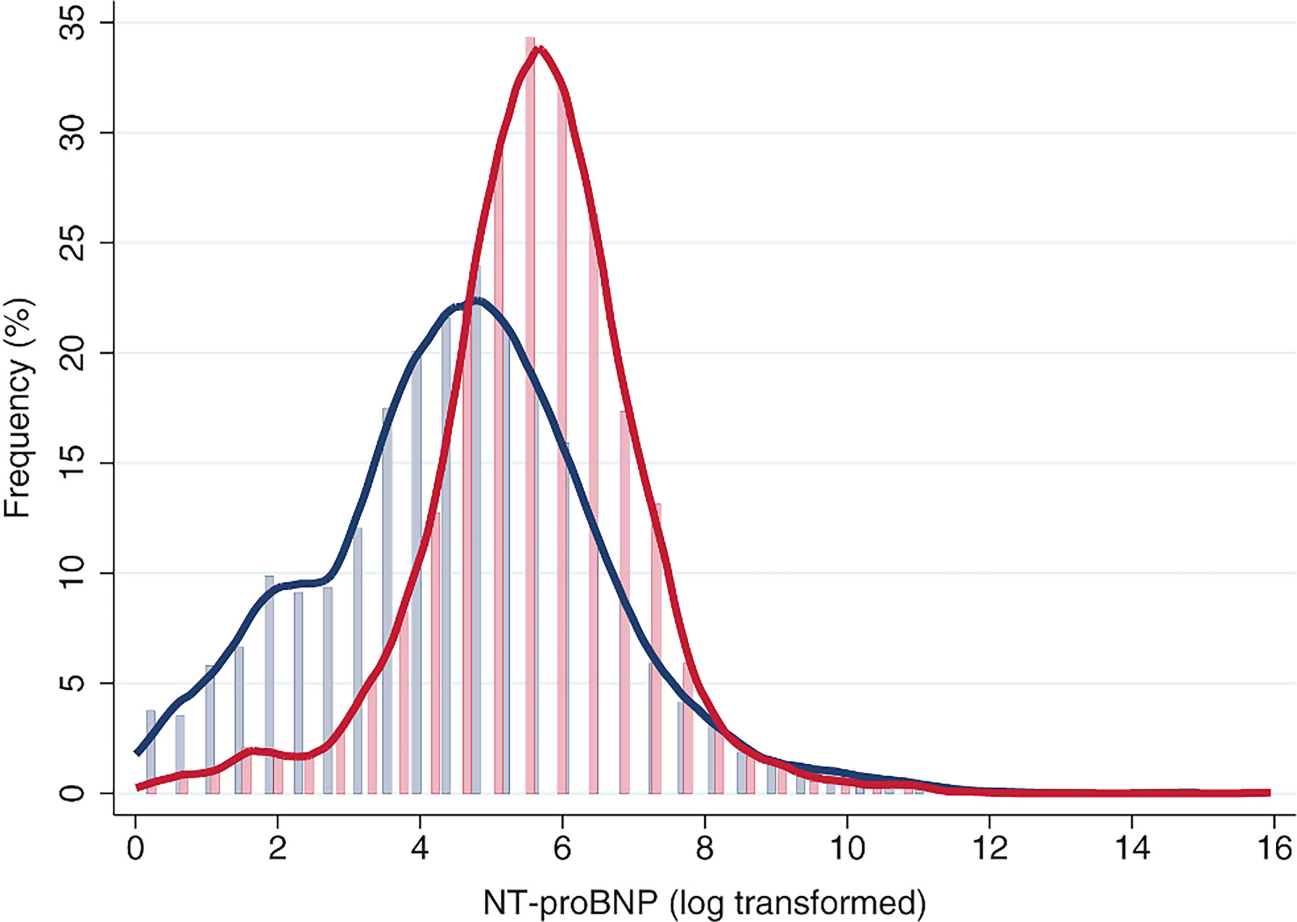
Distribution of N-terminal pro-B-type natriuretic peptide (NT-proBNP) in both sexes. Reproduced with permission from Suthahar et al. (43).

Currently, NT-proBNP is a well-established, powerful biomarker for the diagnosis and prognosis of HF (35–37). It is also a useful biomarker for risk stratification in other several cardiovascular disorders (38, 39). Strong clinical evidence has revealed that several factors influence NT-proBNP levels. Elevated concentrations were observed in patients with various cardiovascular disorders and in patients with renal dysfunction (40, 41). A previous study, which included 7,770 individuals from the Framingham Heart Study and the Malmö Diet and Cancer study, reported that obesity was associated with 6–20% lower NT-proBNP levels, compared to normal-weight status, and insulin resistance was associated with 10–30% lower levels of NT-proBNP, compared to insulin sensitive status (42). Age and sex are also important in modifying circulating levels of natriuretic peptides. Most studies found that at baseline NT-proBNP levels were lower in males than in females (Figure 2) and, in both genders, increases were correlated with age (44).

### Sex Differences in NT-proBNP Levels

Although sex-specific differences in NT-proBNP levels have been documented, the precise mechanism that gives rise to higher NT-proBNP levels in women than in men is not well-established in healthy subjects. Several possible explanations have been explored. One reasonable pathobiological explanation involves the effects of sex hormones. Strong clinical evidence has shown that testosterone could lower cardiac natriuretic peptide levels, probably by upregulating neprilysin activity; this effect might explain why NT-proBNP levels are lower in men than in women (45, 46). Other studies showed that estrogen increased cardiac natriuretic peptide gene expression and its release, which might explain the elevated cardiac natriuretic peptides levels in women compared to men. However, other reports suggested that estrogen also increased neprilysin activity (43, 47). In postmenopausal women, hormone replacement therapy administered for 3 months resulted in elevations in ANP and BNP concentrations (48). Some research however hypothesized that free testosterone could increase lean mass and may directly decrease natriuretic peptide synthesis. This last statement goes beyond the notion that estrogens are primarily responsible for gender differences in natriuretic peptides considering that exogenous estrogen increased the sex hormone-binding globulin with a subsequent lower free testosterone (49). Of note, the profoundly different anthropometric characteristics and fat distributions found in males and females might also play a role in natriuretic hormone levels. Recent evidence from a general population study found that the relationship between NT-proBNP and obesity had a significant sex-associated component. The inverse association between NT-proBNP and obesity was more pronounced among females than among males. Furthermore, among females, but not males, individuals with abdominal (visceral) obesity had lower NT-proBNP levels than individuals with peripheral (subcutaneous) obesity (50). Some studies propose at a molecular level a higher clearance of BNP in obesity due to increased expression of natriuretic peptide receptor on adipose tissue, which binds BNP and leads to its internalization and degradation (51) A reduced release of natriuretic peptides from myocardial tissue in obese individuals have also been pustuled as an alternative hypothesis (52) Therefore, a combination of increased degradation and decreased release may contribute to relative deficiency of natriuretic peptides in obesity.

**Figure 3:**
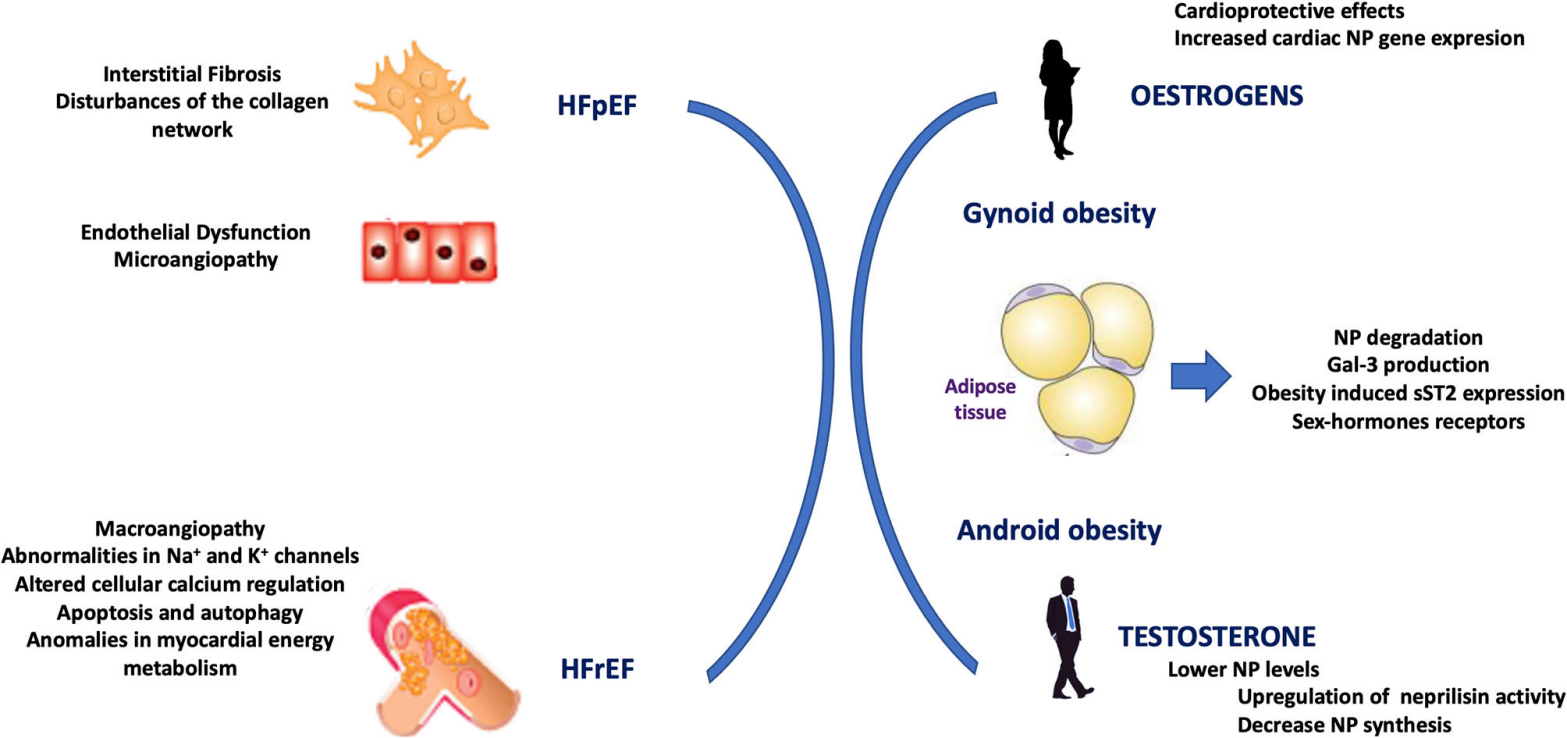
Representation of a lack of coincidences between mechanisms that affect heart failure progression and gender particularities in the context of biomarker levels' variability. HFpEF, heart failure with preserved ejection fraction; HFrEF, heart failure with reduced ejection fraction; NP, natriuretic peptide; Gal-3, Galectine 3; sST2, soluble interleukin-like receptor-like-1.

However, these sex-related dissimilarities observed in the general population appeared to be less pronounced in HF and other disease populations associated with upregulated NT-proBNP levels. Some studies have reported the opposite findings, noting that natriuretic peptide levels were similar or lower in women compared to men (53, 54). However, this change in tendency should be interpreted cautiously, because over the past decade, one of the most robust findings across numerous HF studies was that the gender distribution varied according to the HF phenotype. Among individuals with HF, women significantly outnumber men, and the gender ratio is ~2:1 in HFpEF (6, 20). Numerous reports have shown that natriuretic peptide levels are much lower in patients with HFpEF than in patients with HFrEF (35, 55, 56). Consequently, when studies analyze the convoluted relationship between sex, ejection fraction, and BNP levels in the setting of HF, the results show that women tend to have higher BNP levels than men (57, 58). However, despite the gender-related differences in the levels of natriuretic peptides, the performance of these peptides for diagnosing HF and their prognostic utility are similar in both sexes, and sex specific cut-off points are not usually recommended. At this point, it should also be noted that there is a lack of coincidences between molecular mechanisms that affect HF progression and gender particularities in the context of biomarker levels' variability (Figure 3).

### Prediction of HF Incidence

NT-proBNP levels have shown clinical relevance in predicting the incidence of HF in the general population. High levels were associated with a high risk of HF (59–61), which suggested that elevated baseline levels might reflect subclinical cardiac dysfunction that could subsequently manifest as overt HF. Recent studies have explored sex-specific differences in using NT-proBNP to evaluate cardiac functional competence. Evidence from two community-based studies (44, 61) showed that the optimal cut-off point for detecting moderate to severe left ventricular disfunction was higher in women than in men. The discriminatory ability of the biomarker was similar in both sexes, but the strength of the association might be different between men and women. Indeed, a recent meta-analysis of prospective studies (62) found that NT-proBNP was more strongly associated with incident HF in men than in women. In the near future, the use of natriuretic peptides to assess risk in asymptomatic adults is expected to become translated from clinical studies to routine clinical practice.

## Soluble Interleukin-1 Receptor-Like 1 (ST2)

ST2 is a member of the interleukin-1 receptor family. ST2 exists in both membrane-bound (ST2L) and soluble (sST2) forms. Interleukin-33 (IL-33) is the functional ligand for ST2L, and in the heart, the IL-33/ST2L interaction mitigates cellular responses to mechanical stress. This function is thought to be mediated by the inhibition of apoptosis and cell death (63). Loss of IL-33/ST2L signaling results in unchecked remodeling in the ventricular myocardium, which leads to myocyte hypertrophy, fibrosis, and a decline in left ventricular function (64). In contrast, sST2 acts as a “decoy” receptor for IL-33; thus, sST2 inhibits the cardioprotective effects mediated by the IL-33/ST2L interaction, which indirectly promotes myocardial damage (65). With the development of a highly sensitive ELISA method for measuring sST2 (66), in the last decade, clinical evidence has highlighted the biological and clinical importance of plasma sST2 concentrations. Currently, sST2 is considered a strong, independent prognostic biomarker in patients with myocardial infarction and HF (67, 68).

Clinical data has suggested that sex has a potentially important effect on sST2 concentrations. Women exhibited lower sST2 levels than age-matched men (69). In a large population-based study of ambulatory individuals, women had lower sST2 levels than men, but among older women, an age-associated rise in sST2 concentrations was observed. However, even among older adults, men had higher sST2 levels than women (69). These differences, which seem to be evident beginning in late adolescence (70), were present both in patients with cardiovascular disease and in healthy subjects. Currently, the mechanism underlying these differences has not been elucidated. The hypothesis that sex hormones might be responsible for differences in sST2 levels has not been adequately proven, and current evidence remains controversial. Some studies have supported this hypothesis by showing that elevated testosterone levels were linked to elevated ST2 concentrations, and conversely, exogenous estrogen therapy was linked to lower sST2 levels. In contrast, another study did not find any significant correlation between sex hormones and sST2 levels (69, 71). Obesity is also an important factor to consider in this setting, because sex hormones are produced by adipose tissue, and gender-related differences have been shown in the association between obesity and metabolic diseases. A recent study by Zhao et al. revealed, in an animal model, that obesity induced sST2 expression and secretion in adipocytes (72). A deep physiological understanding of the reasons and clinical relevance of gender-specific differences in sST2 concentrations requires future research.

Due to the prognostic value of ST2 (73–75) and its ability to predict incident HF (76), it has become part of the risk stratification strategy in HF clinical practice guidelines (77). A cut-off point of 35 ng/ml ST2 has been universally adopted as a good indicator of prognosis in both sexes; thus, to date, sex-specific cut-off points have not been needed for risk predictions.

## Galectin-3

Galectin-3 (Gal-3), a unique member of the chimera-type galectins, is involved in a large number of disease processes. It is widely expressed in human tissues, including epithelial, endothelial, and immune cells (78). Gal-3 plays a role in both acute and chronic inflammation, and its effects on cell function include the activation of fibroblasts and macrophages, which lead to fibrosis in various organs, including the heart (79). As a biomarker, Gal-3 has been associated with cardiac function (80); several studies have demonstrated significantly higher Gal-3 levels in patients with HF, particularly those with HFpEF, compared to controls (80). Nevertheless, this biomarker is not predominantly produced in the heart; non-cardiac sources appear to be responsible for high Gal-3 levels in patients with HF (81).

Recent data from population-based studies (82–84) have indicated that plasma Gal-3 levels were slightly higher in women than in men. The physiological explanation for this gender-specific difference is not fully understood, but differences in fat mass might play a role, considering that, for the same body mass index, women typically have 10% more body fat than men (85). Indeed, prior studies have observed an association between total body fat and Gal-3 levels (86). Although the sex-specific prognostic value of Gal-3 in HF remains unknown, baseline Gal-3 concentrations were associated with adverse outcomes during follow-up in patients with acute and chronic HF (87–89). However, the prognostic value of Gal-3 in the setting of chronic HF remains controversial; other biomarkers, such as NT-proBNP or sST2, have frequently exhibited superior predictive value (90). Moreover, other studies have shown that the predictive value of Gal-3 in HF was less pronounced when the analysis was adjusted for renal function (87).

In the Framingham Heart Study, an analysis of more than 3,000 participants showed that elevated Gal-3 concentrations were associated with increases in the risk of new-onset HF (HR 1.28 per 1 standard deviation increase in the log-Gal-3 concentration). This association was clearly attenuated after adjusting for kidney function (82). This “renal implication” highlights the paramount relevance of cardio-renal interactions in the setting of HF, and it suggests that HF might involve a common profibrotic process in the heart and kidneys.

## Less Common Biomarkers in Clinical Practice

In the last decade there has been an intensified interest in additional biomarkers as an objective alternative for diagnosis, prognosis or personalized treatment in HF. Among them is the growth differentiation factor-15 (GDF-15), a member of the transforming growth factor-?? cytokine superfamily with anti-apoptotic, anti-hypertrophic, and anti-inflammatory properties. GDF-15 is weakly expressed in tissues under normal conditions. Although its pathobiology is not fully understood, it is strongly induced by macrophages in response to inflammation and tissue injury. It appears to be only moderately expressed in the heart (81). Despite GDF-15 have been identified as an inflammatory biomarker with prognostic value in several conditions, particularly in cardiovascular diseases (91, 92), with strong association with incident HF (93), sex differences in plasma levels of this biomarker have not been clearly established (94, 95). It has been showed that testosterone together with estradiol significantly decreased GDF-15 levels through an androgren receptor/estrogen receptor-mediated pathway (96). Osteopontin, a glycoprotein expressed in various cell types, including cardiomyocytes and fibroblasts has also gained interest as a prognostic marker in HF. It had been found to be significantly elevated in patients with systolic HF (97). Its cardiac expression promotes myocardial fibrosis and increases left ventricular stiffness (98). It appears that plasma osteopontin levels are higher in men than in women as evidence in the study by Arnlöv et al. (99), however there are lacking evidence in the literature of sex differences in osteopontin expression, and this requires further investigation.

## Conclusions and Perspectives

Most circulating HF biomarkers are used daily by clinicians without taking sex into account. Nevertheless, multiple gender-related differences have been observed in the plasma concentrations of several biomarkers. In the healthy population, women tend to exhibit higher levels of natriuretic peptides and Gal-3 and lower levels of cTn and sST2, compared to men. Plausible biological explanations for these sex-related differences have been postulated, like differences in body composition, fat distribution, or sex hormones. Nonetheless, several clinical studies have shown that these differences were attenuated in patients with HF, despite the fact that distinct gender distributions have been extensively described for different HF phenotypes. Moreover, these sex-related differences do not necessarily translate into a need to use different cut-off points for men and women, either for HF diagnosis or HF prognosis, in clinical practice. Future research should explore the clinical value of considering possible sex-related differences in specific HF biomarkers, in both diagnostic and prognostic settings, with the aim of improving HF management and patient care.

## Author Contributions

GC drafted the manuscript with support from AB-G. All the authors contributed to manuscript revision and read and approved the submitted version.

## Conflict of Interest

AB-G has received speaker fees from Roche Diagnostics. AB-G and JL report a relationship with Critical Diagnostics. The remaining authors declare that the research was conducted in the absence of any commercial or financial relationships that could be construed as a potential conflict of interest.
